# Novel Human Polymorphisms Define a Key Role for the SLC26A6-STAS Domain in Protection From Ca^2+^-Oxalate Lithogenesis

**DOI:** 10.3389/fphar.2020.00405

**Published:** 2020-04-07

**Authors:** Liana Shimshilashvili, Sara Aharon, Orson W. Moe, Ehud Ohana

**Affiliations:** ^1^Department of Clinical Biochemistry and Pharmacology, Faculty of Health Sciences, Ben-Gurion University of the Negev, Beer-Sheva, Israel; ^2^Department of Internal Medicine, University of Texas Southwestern Medical Center, Dallas, TX, United States; ^3^Charles and Jane Pak Center of Mineral Metabolism and Clinical Research, University of Texas Southwestern Medical Center, Dallas, TX, United States; ^4^Department of Physiology, University of Texas Southwestern Medical Center, Dallas, TX, United States

**Keywords:** SLC26A6, citrate, oxalate, kidney stones, NaDC-1

## Abstract

Impaired homeostasis of the carboxylic acids oxalate and citrate, dramatically increases the risk for the formation of Ca^2+^-oxalate kidney stones, which is the most common form of kidney stones in humans. Renal homeostasis of oxalate and citrate is controlled by complex mechanisms including epithelial transport proteins such as the oxalate transporter, SLC26A6, and the citrate transporters, the SLC13’s. These transporters interact *via* the SLC26A6-STAS domain *in vitro*, however, the role of the Sulfate Transporter and Anti-Sigma factor antagonist (STAS) domain in Ca^2+^-oxalate stone formation was not investigated in humans. Here, we report two novel human SLC26A6 polymorphisms identified in the STAS domain of SLC26A6 in two heterozygous carriers. Intriguingly, these individuals have low urinary citrate, but different clinical manifestations. Our *in vitro* experiments indicate that the homolog mutations of SLC26A6(D23H/D673N) and SLC26A6(D673N) alone abolished the expression and function of SLC26A6, and impaired the regulation of SLC13-mediated citrate transport by SLC26A6. On the other hand, the SLC26A6(R621G) variant showed reduced SLC26A6 protein expression and membrane trafficking, retained full transport activity, but impaired the regulation of the citrate transporter. Accordingly, the human SLC26A6(D23H/D673N) carrier showed a dramatic reduction in urinary citrate concentrations which resulted in Ca^2+^-oxalate stones formation, as opposed to the carrier of SLC26A6(R621G). Our findings indicate that the human SLC26A6-STAS domain mutations differentially impair SLC26A6 expression, function, and regulation of citrate transporters. This interferes with citrate and oxalate homeostasis thus potentially predisposes to Ca^2+^-oxalate kidney stones.

## Introduction

The majority of kidney stone formers develop Ca^2+^-oxalate stones, which is a significant health problem that may lead to loss of renal function (Moe, 2006; Evan et al., 2010) and is associated with other morbidities such as hypertension and increased risk of fractures (Borghi et al., 1999; Obligado and Goldfarb, 2008). In fact, more than 85% of kidney stone formers has Ca^2+^-oxalate as part of the stone composition (Evan et al., 2003; Moe, 2006). Ca^2+^-oxalate kidney stones are caused by elevated urinary Ca^2+^ and oxalate, termed hypercalciuria and hyperoxaluria, respectively (Moe, 2006). In addition, low urine concentrations of the Ca^2+^-chelator, citrate, can also contribute to calcium lithogenesis, when coupled to hyperoxaluria, even in the absence of hypercalciuria (Moe, 2006). Sufficient urinary citrate concentrations are crucial to protect against stone formation. We have previously reported that the SLC26A6/NaDC-1 complex of transporters protects against stone formation in a dual fashion (Moe, 2006; Ohana et al., 2013). On one hand, SLC26A6 exclusively mediates oxalate clearance in the intestine and, as a result, *Slc26a6* deletion in mice causes Ca^2+^-oxalate stone formation driven by hyperoxalemia and increased filtered load (Jiang et al., 2006; Knauf et al., 2011). On the other hand, SLC26A6 interacts with the proximal tubule citrate transporter, SLC13A2 or NaDC-1 (sodium dicarboxylate cotransporter-1), to inhibit citrate uptake from the urine. This mechanism controls citrate re-absorption, thus regulating urinary citrate excretion rate and concentrations (Ohana et al., 2013). More specifically, the intracellular STAS domain of SLC26A6 interacts with a specific structural determinant on NaDC-1, namely, the f domain, which is common to all members of the SLC13 transporter family (Khamaysi et al., 2019). Similarly, the STAS domain is located in the intracellular C-terminal of all members of the SLC26 family of transporters (Sharma et al., 2011). Importantly, mutations in or deletion of the entire STAS segment impair SLC26 proteins trafficking to the plasma membrane and their interaction with partner proteins. This underscores the quintessential role that STAS plays in controlling SLC26 function and expression (Ko et al., 2004; Dorwart et al., 2008; Ohana et al., 2013; Geertsma et al., 2015). Remarkably, numerous human mutations were identified in the STAS domain of different SLC26 transporters causing many diseases including, diastrophic dysplasia (SLC26A2) (Cai et al., 2015), congenital chloride diarrhea (SLC26A3) (Dorwart et al., 2008), Pendred syndrome (SLC26A4) (Everett et al., 1997), and infertility (SLC26A8/A3) (Dirami et al., 2013; Rapp et al., 2017; Wedenoja et al., 2017). Notably, the *Slc26a6*/*Nadc-1* complex was shown to control blood pressure by regulating succinate reabsorption at the proximal tubule, which, in turn, regulates the renin-angiotensin system (Khamaysi et al., 2019). This was suggested as one molecular mechanism that underlies the association between hypertension and kidney stone formation (Borghi et al., 1999; Cappuccio et al., 1999; Obligado and Goldfarb, 2008). Several SLC26A6 polymorphisms were identified in Ca^2+^-oxalate stone formers, however, the vast majority of the polymorphisms are located in the catalytic transmembrane domain (Corbetta et al., 2009; Lu et al., 2016). For example, the SLC26A6(V206M) polymorphism, which we also found in our cohort, was shown to be associated with kidney stones development and primary hyperparathyroidism patients (Monico et al., 2008; Corbetta et al., 2009). Here, we report two novel polymorphisms in the STAS domain of SLC26A6 found in two individuals. One compound polymorphism (D23H/D673N) was identified in a Ca^2+^-oxalate stone former. The other polymorphism, R621G, was identified in an individual that did not have clinically detectable stones to date. Identification of the mechanism that leads to these different clinical outcomes will help delineate the role that the regulatory SLC26A6-STAS domain plays in controlling citrate/oxalate homeostasis and modifies Ca^2+^-oxalate lithogenic propensity. Therefore, we pose the question: What is the mechanism by which SLC26A6-STAS domain polymorphisms impair citrate homeostasis that may lead to Ca^2+^-oxalate stone formation?

## Materials and Methods

### Clinical Studies

Stone-formers were recruited from the Mineral Metabolism Clinic at the Pak Center of Mineral Metabolism and Clinical Research at the University of Texas Southwestern Medical Center. Healthy non-stone formers were recruited from the staff and students on campus with a protocol approved by the University of Texas Southwestern Institutional Review Board and informed consent was obtained from each of the participating subjects. Subject characteristics are shown in **Table 1**. Calcium stone formers all had stone analysis showing 70–100% calcium oxalate in the stone samples. Outpatient 24 h urines were collected on random *ad lib* outpatient diets and urinary stone risk profile was assayed by the Clinical Laboratory Improvement Amendments (CLIA)-certified laboratory and genotyping was performed by the Sequencing Core at the Pak Center of Mineral Metabolism and Clinical Research (Reed et al., 2002). The clinical chemistry methods are standard, in particular, citrate was measured enzymatically by citrate lyase (Cobas Fara, Roche, NJ), creatinine by picric acid method (Olympus AU400), and oxalate by ion chromatography (Dionex, Sunnyvale, CA).

**Table 1:** Demographics of human study subjects. Caucasian stone-formers and healthy non-stone formers were recruited as described in the *Materials and Methods* section. Outpatient 24 h urines were collected on random *ad lib* outpatient diets and urinary stone risk profile was assayed by the Clinical Laboratory Improvement Amendments (CLIA)-certified laboratory and genotyping was performed by the Sequencing Core at the Pak Center of Mineral Metabolism and Clinical Research (Reed et al., 2002).

	Non-stone formers			Stone formers		
	WT	R621G–Het	V206M–Het	WT	D23H–Het D673N–Het	V206M–Het
Ethnicity (NH/H)*	20/1	1/0	2/0	18/0	1/0	8/1
Gender (M/F)	7/14	1/0	1/1	13/5	0/1	7/2
Age, years	45 ± 11	33	50 ± 7	44 ± 14	43	43 ± 14
(Min–max)	(20–57)		(45–55)	(8–61)		(18–61)

*All subjects are Caucasian.

### Plasmid Constructs

The plasmids used were the human SLC26A6 clone (NM_022911) in the pCMV6-AC vector or pCMV6-AC-mkate vector and the human NaDC1 clone (BC096277) in the pCMV6-AC-Myc-His vector. Site-directed mutants were generated with the QuikChange Lightning Mutagenesis Kit (Agilent, Santa Clara, CA) and the appropriate primers. The products were verified by Sanger sequencing (Hylabs).

### Intracellular pH Measurements and Fluorescent Images Acquisition

Intracellular pH was measured using a single cell real-time imaging system equipped with anEclipse Ti inverted microscope (Nikon, Japan), PE-4000 LED monochromator (CoolLEd, UK), andHamamatsu Flash 4.0LT camera (Hamamatsu photonics, Japan). This system was also utilized foracquisition of the mKate images in **Figure 4C**. HEK293 cells, transiently expressing either SLC26A6(WT), or SLC26A6(R621G), SLC26A6(D674N), SLC26A6(D23H/D674N) and an empty vector as a control, were seeded on coverslips (D674 is the homolog of D673 in SLC26A6 isoform No. 1; NCBI accession No. NP_075062.2). Cells were stained on stage using 2 μM BCECF-AM (Biotium Inc, CA) and signal was measured ratiometrically, using excitation wavelength of 490 nm *versus* 435 nm and detected at 530 nm. Cells were incubated with BCECF at room temperature for 5 min and washed with regular solution [prepared with 10 mM 4-(2-hydroxyethyl)-1-piperazineethanesulfonic acid (HEPES), 10 mM glucose, 140 mM NaCl, 5 mM KCl, 1 mM MgCl_2_, 1 mM CaCl_2_, and pH was adjusted to 7.4)] for at least 10 min until stabilization of the fluorescent signal. Subsequently, the cells were perfused with regular solution until establishment of a base line. Next, regular solution was replaced with a HCO_3_^−^ buffered solution (regular solution was adjusted to 120 mM NaCl, 25 mM NaHCO_3_^−^, and 2.5 mM HEPES) or, with a Cl^−^ free solution (HCO_3_^−^ buffered solution was papered with gluconate to replace Cl^−^). All HCO_3_^−^ buffered solutions were gassed with 5% CO_2_ and 95% O_2_.

### Immunoblot and Co-Immunoprecipitation

HEK293T cells were transfected with the indicated plasmids and after 2 days, lysates were prepared [lysis buffer contained phosphate-buffered saline (PBS), 10 mM Na^+^-pyrophosphate, 50 mM NaF, and pH was adjusted to 7.4. 1 mM Na^+^-orthovanadate, 1% Triton X-100, and a cocktail of protease inhibitors (Roche) were freshly added before each use]. The cells were placed on ice and scraped after addition of lysis buffer. Protein extracts were incubated with Protein G Sepharose beads (Sigma-Aldrich, St. Louis, MO) and anti-His antibody (1:100) (Thermo Fisher Scientific, Waltham, MA) overnight at 4°C. The beads-protein complexes were incubated for 4 h at 4°C, centrifuged, and washed with lysis buffer four times. Samples were prepared for running on sodium dodecyl sulfate polyacrylamide gel electrophoresis (SDS-PAGE) by heating (37°C for 30 min) in SDS sample buffer. Subsequently, the samples were transferred to nitrocellulose membranes and incubated overnight at 4°C with anti-SLC26A6 (1:500) (ab 172684, Abcam) and the next day exposed to the appropriate secondary antibody. Signal was developed using enhanced-chemiluminescence (ECL) substrate (CYANAGEN).

### Cell Surface Expression

Surface expression was analyzed using biotinylation assay. HEKT cells transfected with the appropriate plasmids were incubated with 0.5 mg/ml EZ-LINK Sulfo-NHS-LC-Biotin (Thermo Fisher Scientific, Waltham, MA) for 30 min on ice. The biotin was quenched using 50 mM glycine and lysates were prepared as previously described. Total protein concentration was assessed by BCA method. The lysates were incubated with Neutravidin agarose resin (Thermo Fisher Scientific, Waltham, MA) for 2 h at 4°C and washed three times using lysis buffer. Protein were isolated by adding 50 μl sample buffer and western blot analysis was performed as previously described. The blots were analyzed after incubation of the membranes overnight either with anti-SLC26A6, anti-tRFP (Evrogen) antibodies, or anti-β-actin (Sigma-Aldrich, St. Louis, MO) antibody for 1 h at room temperature.

### Succinate Uptake Measurements

Succinate is an established substrate for transport by NaDC-1 and is an accepted surrogate for citrate. HEK293T cells were transfected with the indicated plasmids and washed with PBS (biological industries). Subsequently, an incubation solution containing 5 mM KCl, 10 mM HEPES, 10 mM glucose, and 140 mM NaCl (pH adjusted to 7.4) was supplemented with 1 mM Na^+^ succinate and 1 mCi ^14^C succinic acid (ViTrax, Inc., Fullerton, CA) per 1.6 mM cold succinate and added to the cells. The cells were then washed twice with incubation solution and 0.5 ml NaOH (1M) was immediately added to lyse the cells. The lysates were then transferred to scintillation vials containing 0.250 ml of HCl (2M). Finally, radioactivity was determined by liquid scintillation counting using a Packard 1900CA TRI-CARB analyzer.

### 3D Protein Model Prediction

The putative structure of SLC26A6 (NCBI accession No. NP_075062.2) was predicted using HHPred software (Soding et al., 2005) with high homology to the cryo-EM structure of SLC26A9 (PDB_ID: 6RTC) (Walter et al., 2019). Prediction parameters: probability=100, E-value = 2.2e−85, score=774.77, identities=39%, similarity=0.742, SS = 54.8, Cols = 632, length = 643. The final model was generated and visualized using PyMOl software (Schrödinger, Germany) (Pettersen et al., 2004).

## Results

### Two Human SLC26A6-STAS Domain Polymorphisms Have Different Clinical Manifestations

**Figure 1:**
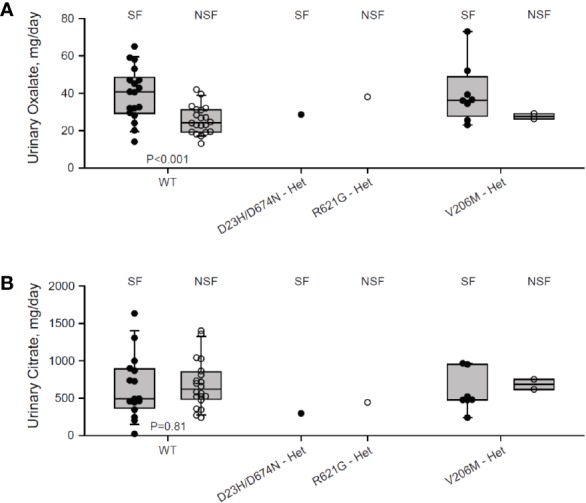
Two individuals carrying heterozygous SLC26A6-STAS domain polymorphisms show hypocitraturia. We monitored urinary oxalate **(A)** and citrate **(B)** concentrations in hyperoxaluric stone formers (SF) and non-stone formers (NSF) that carry the V206M, D23H/D673N, R621G polymorphisms, or WT. P values by ANOVA are indicated. It is noteworthy that both hyperoxaluria and hypocitraturia are risk factors in the general Ca^2+^-oxalate SF population but cohort who underwent genotyping was biased for hyperoxaluric patient and hence hypocitraturia was “selected out”.

We genotyped a cohort of 27 Ca^2+^-oxalate kidney stone formers and 23 healthy non-stone formers, and identified two SLC26A6 polymorphisms located in the region that encodes for the intracellular STAS domain (**Supplementary Figure 1**). Interestingly, one compound polymorphism, SLC26A6(D23H/D673N), was found in a kidney stone former, while the other, SLC26A6(R621G), was found in a non-stone former. We compared the urinary oxalate and citrate concentrations of these individuals to either SLC26A6(WT) or SLC26A6(V206M) polymorphism carriers, which were either healthy (non-stone formers) or stone-formers, as indicated. Interestingly, stone formers who carry the catalytic transmembrane domain polymorphism, SLC26A6(V206M), showed a trend of higher urinary oxalate levels compared to non-stone formers, which was not statistically significant due to the low number of SLC26A6(V206M) carriers in our cohort (**Figure 1A**). However, the SLC26A6(V206M) carriers citrate concentrations were not different (**Figure 1B**). Moreover, the SLC26A6(V206M) urinary oxalate and citrate values were similar to WT in both healthy and stone forming patients (**Figures 1A, B**). Unexpectedly, the (R621G) carrier did not develop kidney stones to our knowledge, in spite of high urinary oxalate and relatively low citrate compared to both WT and V206M individuals. This subject left our institution soon after the original assessment and it is plausible that kidney stones formed later and did not enter our record. However, the individual with (D23H/D673N) polymorphism had normal urinary oxalate levels, but dramatically low urinary citrate compared to all other groups. Remarkably, only three other stone formers in our cohort reached citrate values as low or lower than the D23H/D673N polymorphism carrier. These findings suggest that, in humans, point mutations and polymorphisms in the SLC26A6-STAS domain can interfere with citrate/oxalate homeostasis, but may be not be sufficient to lead to frank disturbances in urinary chemistry or Ca^2+^-oxalate stone formation. Nevertheless, the level of urinary citrate concentrations obtained as an outpatient on a random diet is unlikely sufficient for determining the risk of stone formation.

### The Transport Function of SLC26A6(D673N) Homolog Mutant Is Abolished While the Activity of SLC26A6(R621G) Is Retained

**Figure 2:**
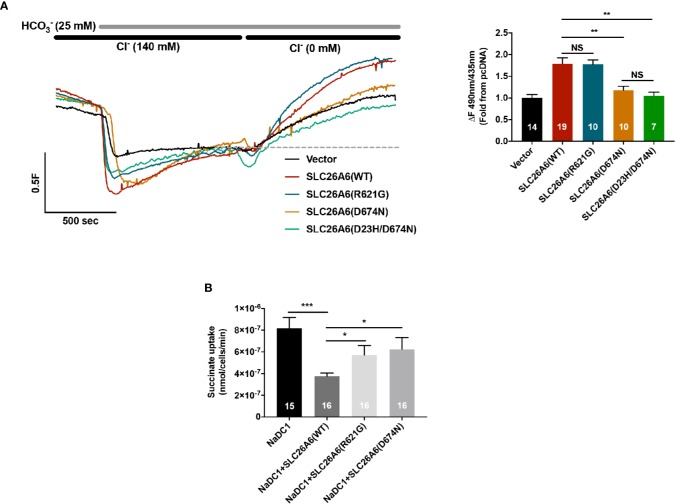
Functional and regulatory properties of SLC26A6 are compromised by the sulfate transporter and anti-sigma factor antagonist (STAS) domain polymorphisms (D23H/D673N) and (R621G). **(A)** Representative traces and summary of the human SLC26A6 Cl^−^/HCO_3_^−^ exchange activity monitored in cells transfected with either an empty vector (control), SLC26A6(WT), SLC26A6(D674N), SLC26(D23H/D674N), or SLC26A6 (R621G), as indicated. Transport activity was monitored as the fluorescence change from the new baseline (dashed line) to peak after prefusion with 0 Cl^−^. **(B)** NaDC-1-mediated succinate uptake was monitored using a radiolabeled ^14^C-succinate flux assay in cells expressing NaDC-1 in the presence or absence of WT or mutant SLC26A6, as indicated. The background signal monitored in control cells (transfected with and empty vector) was subtracted. *P < 0.05, **P < 0.01, ***P < 0.001, NS = P > 0.05.

As previously reported, SLC26A6 controls oxalate clearance in the intestine and also regulates citrate reabsorption in the kidney proximal tubule (Jiang et al., 2006; Ohana et al., 2013). Either of these functions or both are crucial to control oxalate/citrate homeostasis and modify stone risk. To test whether the STAS domain polymorphisms SLC26A6(R621G) and SLC26A6(D23H/D673N) affect the function of SLC26A6, we generated an SLC26A6(R621G) as well as SLC26A6(D23H/D674N) and SLC26A6(D674N) point mutations. Notably, D674 is the homolog of D673 in SLC26A6 isoform No. 1 (NCBI accession No. NP_075062.2). Therefore, the only mutation used for the *in vitro* studies is D674N or the double mutant D23H/D674N, which are equivalent to D673N and D23H/D673 in the isoform we used. Next, we monitored SLC26A6-mediated Cl^−^/HCO_3_^−^ exchange activity in HEK293 cells expressing either SLC26A6(WT) or mutants. As shown in **Figure 2A**, while the transport function of the SLC26A6(D23H/D674N) double mutant (and SLC26A6(D674N)) is completely abolished, the function of SLC26A6(R621G) is fully retained indicating the completely different biologic consequences of these base changes. Notably, these findings suggest that SLC26A6 function is in correlation with the clinical manifestation described in **Figure 1**; showing that the individual with SLC26A6(D23H/D673N) polymorphism forms kidney stones, while the individual with SLC26A6(R621G) does not.

### The Sulfate Transporter and Anti-Sigma Factor Antagonist Domain Polymorphisms Hamper SLC26A6 Mediated Inhibition of the Citrate Transporter, NaDC-1

The essential function of the SLC26 transporters STAS domain in the interaction and regulation of partner proteins, including NaDC-1, suggests that mutations in this protein region may affect SLC26A6 interaction with and regulation of NaDC-1. To address this, we monitored NaDC-1 function by measuring succinate uptake into HEK293 cells expressing NaDC-1 alone or in the presence of either SLC26A6(WT) or mutants. Our results indicate that SLC26A6 significantly inhibits NaDC-1, yet, both STAS mutations dramatically impair the ability of SLC26A6 to inhibit NaDC-1 (**Figure 2B**). This suggests that the SLC26A6-STAS mutations affect either the interaction with NaDC-1, the regulation of NaDC-1 by SLC26A6, or both.

### The STAS Domain Polymorphisms Impair SLC26A6 Expression

**Figure 3:**
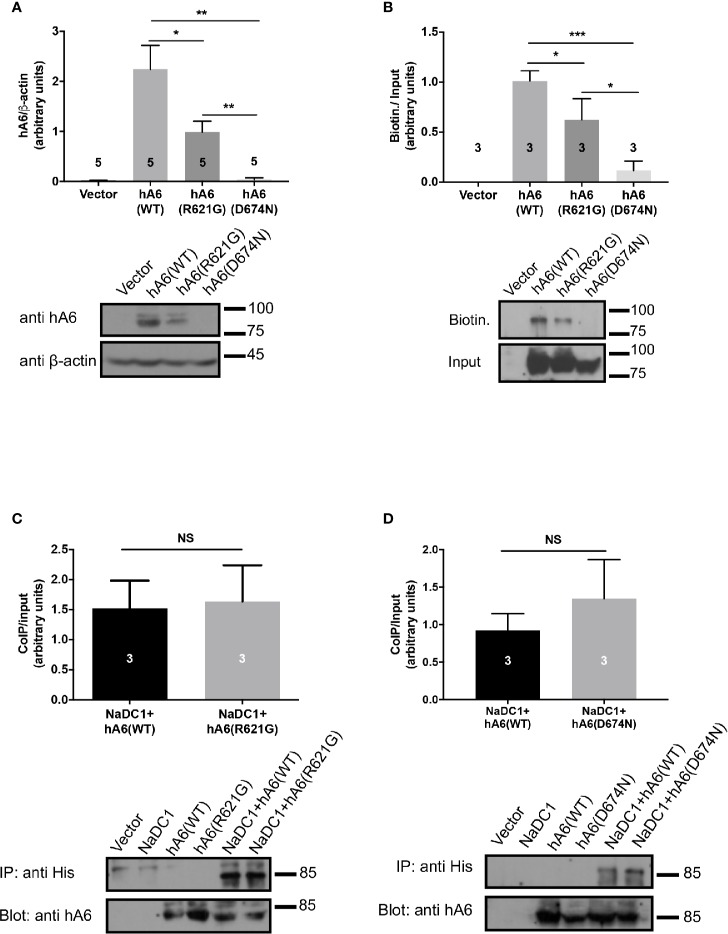
The sulfate transporter and anti-sigma factor antagonist (STAS) domain mutations impair protein expression, trafficking to the plasma membrane but not the interaction with NaDC-1. **(A)** The expression of human SLC26A6 (hA6) monitored in lysates of HEK293T cells transfected with an empty vector SLC26A6(WT), SLC26A6(D674N), or SLC26A6 (R621G) compared to β-actin expression. **(B)** The membrane expression of the indicated proteins was monitored using a biotinylation assay after adjustment of the total protein levels as shown in the input blot. The interaction between NaDC-1, SLC26A6, and mutants was monitored using a co-immunoprecipitation (Co-IP) assay. The western blot analyses in **(C, D)** indicate that the SLC26A6-STAS domain mutations retain interaction with NaDC-1. *P < 0.05, **P < 0.01, ***P < 0.001, NS = P > 0.05.

To explain the different functional effects of the human polymorphisms, we transfected HEK293 cells with either SLC26A6(WT), SLC26A6(D674N), or SLC26A6(R621G) and monitored the expression by immunoblot. As presented in **Figure 3A**, we found that the total cellular expression of SLC26A6(R621G) was higher than SLC26A6(D674N), but both were significantly lower than WT. This indicates that both mutations down-regulate the total expression of SLC26A6 protein. Next, we aimed to test whether the reduced expression also affects the trafficking of the mutants to the plasma membrane. To this end, we monitored the cell surface expression of either WT or mutant SLC26A6 proteins using a surface biotinylation assay after adjusting the input protein levels. We found that the membrane expression of D674N is dramatically low compared to R621G and WT (**Figure 3B**). This explains both the nearly abolished function of this mutant and corresponds to the failure to inhibit NaDC-1 transport by the D674N mutant. Nonetheless, the membrane expression of R621G is also significantly lower than WT (**Figure 3B**), but apparently this level of R621G expression is sufficient to retain full SLC26A6 function.

### The Interaction of Both D674N and R621G With NaDC-1 Is Similar to SLC26A6(WT)

As shown in **Figure 1**, the carriers of both mutants exhibit low urinary citrate concentrations. Therefore, we hypothesized that the mutations may interfere with SLC26A6-NaDC-1 interaction, which is mediated by the STAS domain. To this end, we tested the level of interaction between NaDC-1 and SLC26A6 mutants compared to WT utilizing co-immunoprecipitation (Co-IP), following adjustment of SLC26A6 expression levels. As shown in **Figures 3C, D**, the interaction level of both D674N and R621G with NaDC-1 is similar to the interaction between NaDC-1 and SLC26A6(WT). This may suggest that the mutations do not interfere with NaDC-1 interaction, or that the STAS domain region that encompasses R621 and D674 does not mediate SLC26A6-NaDC-1 binding.

Together, our results, thus far, indicate that D674N (which corresponds to the human polymorphism D673N) dramatically impairs the expression of SLC26A6, and abolishes the trafficking of SLC26A6 to the plasma membrane and, consequently, hampers SLC26A6 transport function. On the other hand, the R621G mutant lowers SLC26A6 total expression and trafficking, however, the residual expression is sufficient to retain full transport activity. Notably, the low surface expression of both mutants relative to WT is insufficient to inhibit NaDC-1, since NaDC-1 inhibition is eliminated by D674N and R621G (**Figure 2B**).

### Potential Rescue of SLC26A6(D674N) Membrane Expression

**Figure 4:**
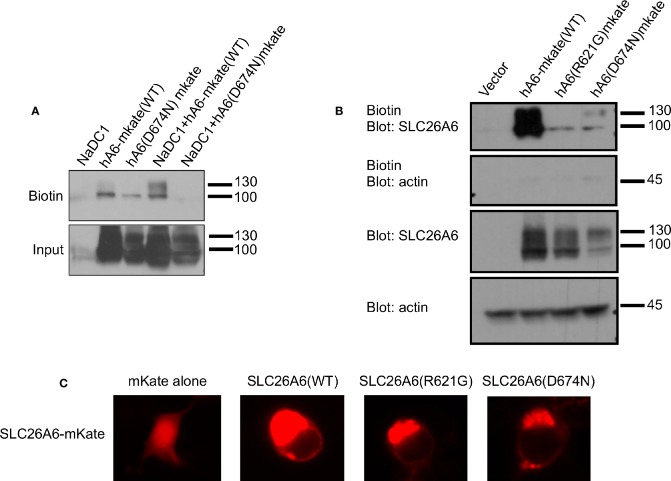
The membrane expression of SLC26A6(D674N) is recovered by the mKate tag, but not by NaDC-1 expression. We monitored human SLC26A6 (hA6) trafficking to the plasma membrane by biotinylation using similar total protein concentrations (no adjustment). As shown in **(A)**, in the presence of NaDC-1, the expression of slc26a6 was even lower than in the absence of NaDC-1. However, the mKate tagged SLC26A6(D674N)mKate protein showed membrane expression similar to that of SLC26A6(R621G)mKate **(B)**. The images in **(C)** describe the cellular distribution of the indicated mKate tagged proteins compared to the cytoplasmic distribution of mKate alone.

The trafficking of transport proteins to the membrane may be rescued by partner proteins that form complexes in the membrane, as demonstrated for CFTR (Bosch and De Boeck, 2016). Therefore, we monitored the membrane expression of SLC26A6(D674N) in the presence of NaDC-1. The results in **Figure 4A** suggest that the surface expression of the D674N mutant is not improved and even hampered by NaDC-1 expression. However, monitoring the membrane expression of the same mutant with a fluorescent protein tag at the C-terminus increased the protein membrane expression (**Figures 4B, C**). This suggests that adding a soluble peptide to the STAS domain slightly compensates for the trafficking impairment caused by the STAS(D674N) mutant. Therefore, the SLC26A6-STAS domain is a potential therapeutic target for diseases caused by impaired trafficking of SLC26 transporters.

## Discussion

Many factors increase the risk for kidney stone formation, including, abnormal urine pH, high urine calcium, high urine oxalate, and low concentrations of the major urine Ca^2+^ buffer, citrate. Elevated urine oxalate coupled with low urinary citrate is a dire combination and imposes a major risk for Ca^2+^-oxalate kidney stones formation, even in the absence hypercalciuria (Moe, 2006; Moe and Preisig, 2006). Interestingly, the homeostasis of oxalate and citrate is controlled by the SLC26A6/NaDC-1 complex of transport proteins (Ohana et al., 2013). SLC26a6 and NaDC-1 reciprocally regulate their function—SLC26A6 strongly inhibits NaDC-1, while NaDC-1 slightly activates SLC26A6 (Ohana et al., 2013). These are teleogically logical interactions that control the luminal concentrations of citrate (anti-lithogenic) and oxalate (pro-lithogenic). This occurs due to interaction between the SLC26A6-STAS and the NaDC-1-H4c domains (Ohana et al., 2013; Khamaysi et al., 2019). However, the role of the SLC26A6-STAS domain in human Ca^2+^-oxalate stone formation has not been explored and is poorly understood. In the current study, we report of two novel SLC26A6 polymorphisms found in two different individuals. Intriguingly, one individual that carries the heterogeneous single nucleotide polymorphism, R621G, has high urinary oxalate and low urinary citrate, but has not form kidney stones to date. The other individual was diagnosed with Ca^2+^-oxalate kidney stones showing significant hypocitraturia with normal urinary oxalate concentrations (**Figure 1**). We studied the role of the STAS domain mutations *in vitro*, and found that the D674N mutation (which is homologous to D673N) abolished SLC26A6 expression, trafficking, transport function, and regulation of the citrate transporter, NaDC-1. Although the patient carries a compound D23H/D673N polymorphism, we focused on the STAS domain missense mutation D673N (we tested the homologous mutant D674N), which impaired protein expression even in the absence of D23H (**Figure 3A**). These findings are in agreement with the clinical manifestation of Ca^2+^-oxalate kidney stone formers, since impaired SLC26A6 function hampers the exclusive intestinal oxalate clearance pathway as well as proximal tubule citrate transport regulation (Aronson, 2010). Indeed, the D23H/D673N carrier had lower citrate concentrations compared to other stone formers (**Figure 1B**). Yet, the normal urinary oxalate concentrations in D23H/D673N carrier are unexpected and require further investigation (**Figure 1A**).

On the other hand, the heterogeneous polymorphism R621G impaired SLC26A6 expression and trafficking, but to a lesser extent compared to D674N (**Figures 3A, B**). Consequently, the SLC26A6 activity was retained, however, the membrane expression was not sufficient to preserve the NaDC-1 inhibition by SLC26A6, which was largely abolished (**Figures 2A, B**). Nonetheless, the R621G carrier showed hyperoxaluria and relatively low urinary citrate, which was less dramatic compared to that of the D673N carrier (**Figures 1A, B**).

We present findings in humans and *in vitro*, that the SLC26A6-STAS domain plays a key role in controlling citrate homeostasis. Based on our previous reports, we suggest that specific STAS domain mutations are expected to cause low urinary citrate due to impaired inhibition of SLC13-mediated citrate transport and subsequent elevated citrate absorption. This would potentially lower urinary citrate as we, indeed, measured in urine samples that were collected from STAS-polymorphism carriers (**Figure 1**). Notably, our results suggest that the extent of urinary citrate reduction may contribute to the clinical outcome. The D23H/D673N polymorphism carriers had a dramatic reduction in urinary citrate compared to other stone formers that likely induced lithogenesis even in the absence of hyperoxaluria. Nevertheless, the citrate concentrations monitored in urine samples of the R621G carrier were 50% higher compared to the D23H/D673N carrier, who also had high urinary oxalate, but did not develop stones (**Figures 1A, B**). This may indicate that a substantial decrease in urinary citrate caused by impaired SLC26A6 regulation of citrate absorption is sufficient to cause stone formation even in the absence of hyperoxaluria. These observations are rather intriguing but the clinical conclusions are limited since we identified only one individual that carries either of the polymorphisms in our cohort. Finally, our previous report indicated that in mice *Slc26a6*\*Slc13* complex also controls blood pressure by regulating succinate homeostasis (Khamaysi et al., 2019). Hence, the effects of impaired SLC26A6-STAS domain function may extend beyond kidney stone formation and could also lead to hypertension, which is, indeed, strongly associated with kidney stones (Obligado and Goldfarb, 2008).

Numerous studies by others and us have shown that the STAS domain is essential for transporter trafficking and regulation of partner proteins *via* interaction (Sharma et al., 2011). As a result, human mutations within the STAS domain of many SLC26 family members can cause diseases (Rapp et al., 2017). For example, a specific STAS domain mutation in SLC26A3, which is associated with male subfertility, impairs SLC26A3 interaction with CFTR (Wedenoja et al., 2017). Another study showed that a specific SLC26A2-STAS mutation associated with the skeletal disease, diastrophic dysplasia, impairs SLC26A2 trafficking to the plasma membrane (Rapp et al., 2017). Therefore, a potential therapeutic strategy could be rescuing the mutant SLC26A2 surface expression. Remarkably, a similar therapeutic strategy underlies the cystic fibrosis treatment by Lumacaftor^®^ that increases surface expression of mutated CFTR channels (Van Goor et al., 2011). Our results in **Figures 4B, C** suggest that a C-terminal fluorescent tag may improve the membrane trafficking of SLC26A6(D674N), however, further analysis is required to test the effects of the tag on STAS domain structure and function. Our findings indicate that SLC26 transporters trafficking or protein-protein interactions caused by STAS domain mutations may be rescued. In the future, the screening and development of compounds that specifically target different SLC26 STAS domains and regions may increase trafficking or correct regulatory defects to treat SLC26 related diseases including kidney stones and hypertension.

## Data Availability Statement

The raw data supporting the conclusions of this article will be made available by the authors, without undue reservation, to any qualified researcher.

## Ethics Statement

The studies involving human participants were reviewed and approved by The University of Texas Southwestern Institutional Review Board. The patients/participants provided their written informed consent to participate in this study.

## Author Contributions

EO designed the study. LS performed experiments. SA generated clones. EO, LS, and OM were involved in data analysis. Human data acquisition was performed by OM. Funding was acquired by EO and OM. EO, LS, and OM wrote the manuscript. All authors contributed to critical revision of the manuscript and approved the final version.

## Funding

This work was supported by BSF grant (No. 2015003) to EO and by the Israel Science Foundation grants No. 271/16 and 2164/16 to EO. OM was supported by the National Institutes of Health R01DK081423, R01 DK115703, R01 DK091392, and P30 DK079328) and the Charles Pak Foundation.

## Conflict of Interest

The authors declare that the research was conducted in the absence of any commercial or financial relationships that could be construed as a potential conflict of interest.

## Supplementary Material

Supplementary Figure 1 A putative 3D model of SLC26A6 and multiple sequence alignment (MSA) of human isoforms. We generated a putative 3D model of the human SLC26A6 based on the cryo-EM model reported for the SLC26A9 transporter. The model was generated as described in the methods section. The model shows the SLC26A6 transmembrane domain (green), the STAS domain (yellow) and the D674 residue location (red). The model lacks the IVS region of STAS (dashed line) which is flanked by the blue residues and harbors R621. The MSA analysis shows R621 (dark grey) and D674 (red) conservation between different human SLC26A6 isoforms. The IVS residues of human SLC26A6 isoform 1, which was used in our study, are highlighted in light grey.
