# Supplementary figures and images for: Novel Human Polymorphisms Define a Key Role for the SLC26A6-STAS Domain in Protection From Ca2+-Oxalate Lithogenesis
Source: Front Pharmacol. 2020 Apr 7;11:405. doi: 10.3389/fphar.2020.00405 (PMC7154107; doi:10.3389/fphar.2020.00405)

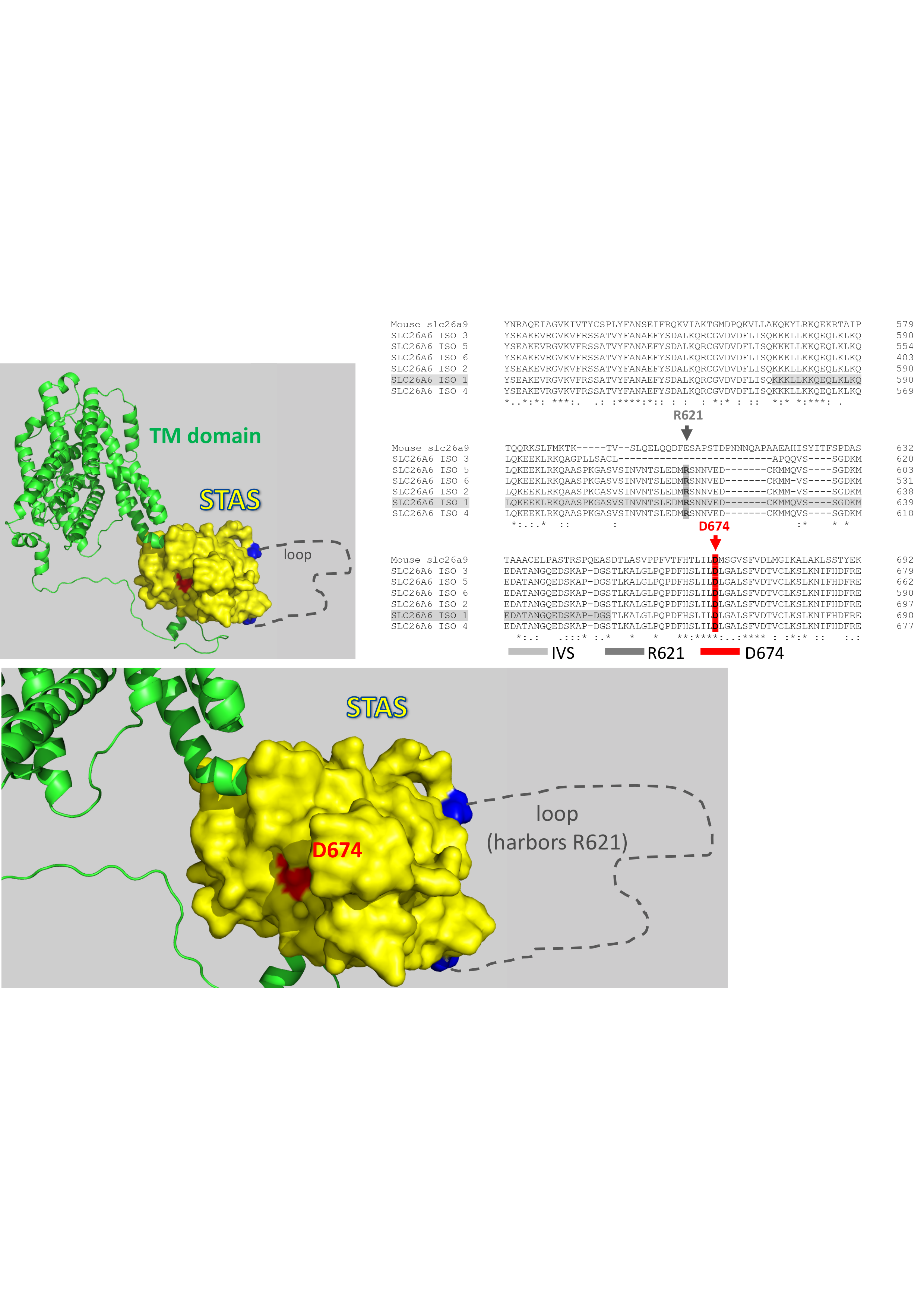

Supplement: Supplementary Figure 1 — A putative 3D model of SLC26A6 and multiple sequence alignment (MSA) of human isoforms. We generated a putative 3D model of the human SLC26A6 based on the cryo-EM model reported for the SLC26A9 transporter. The model was generated as described in the methods section. The model shows the SLC26A6 transmembrane domain (green), the STAS domain (yellow) and the D674 residue location (red). The model lacks the IVS region of STAS (dashed line) which is flanked by the blue residues and harbors R621. The MSA analysis shows R621 (dark grey) and D674 (red) conservation between different human SLC26A6 isoforms. The IVS residues of human SLC26A6 isoform 1, which was used in our study, are highlighted in light grey. [file Image_1.tif]
